# Carbonation and Phase Evolution in MgO-SiO_2_ Cements: Impact on Strength

**DOI:** 10.3390/molecules30051072

**Published:** 2025-02-26

**Authors:** Gonzalo Mármol, Ana Fernández-Jiménez, María-Teresa Blanco-Varela, Inés García-Lodeiro

**Affiliations:** Eduardo Torroja Institute for Construction Science (IETcc-CSIC), Serrano Galvache, 4, 28033 Madrid, Spain; anafj@ietcc.csic.es (A.F.-J.); iglodeiro@ietcc.csic.es (I.G.-L.)

**Keywords:** M-S-H cement, carbonation, hydrated magnesium hydroxycarbonates, pore refinement, CO_2_ encapsulation

## Abstract

Magnesium silicate hydrate (M-S-H) binders, synthesized from magnesia and silica, exhibit promising mechanical and thermal properties but face challenges in early strength development due to delayed kinetics and limited MgO solubility. This study investigates the impact of early exposure to CO_2_-saturated atmospheres on MgO-SiO_2_ cementitious systems, emphasizing the role of carbonation in phase evolution and mechanical performance. Early carbonation promotes the formation of hydrated magnesium hydroxycarbonates (HMHC), altering hydration pathways and reducing M-S-H gel content. Key analyses, including XRD, TGA, SEM-EDS, and FTIR, reveal that higher carbonation levels correlate with reduced Mg(OH)_2_ stability at early ages, an enhanced precipitation of HMHC phases, and significant effects on mineralogy and strength. Results underscore the influence of formulation, water-to-cement ratio, and early carbonation in optimizing strength and phase development, providing a pathway to more efficient MgO-SiO_2_ cement systems with reduced reliance on reactive SiO_2_.

## 1. Introduction

Inorganic binders based on MgO and SiO_2_ undergo hydration reactions, upon mixing with water, forming hydrated magnesium silicates with low crystallinity, known as M-S-H gels. These gels, which are nearly amorphous and represent low crystallinity phases [1,2], offer potential as sustainable alternatives to Portland cement due to their low or even negative CO_2_ emissions when derived from magnesium silicate-rich minerals [3,4]. M-S-H gels are also considered for use as lower-pH binders, suitable for applications such as nuclear waste encapsulation in the pH range of 8 to 10 [5,6,7,8]. Additionally, the lower alkalinity of the pore solution in M-S-H binders makes them effective as matrices for natural fibers, enhancing their utility in reinforcement applications [9,10]. These systems also exhibit high thermal endurance, good surface gloss, lightweight properties, and excellent mechanical characteristics [8,11].

In MgO-SiO_2_ systems, the initial reaction upon mixing the powdered material with water involves MgO reacting to form Mg(OH)_2_. Due to the low solubility of Mg(OH)_2_, only a small amount dissociates, releasing Mg^2+^ ions and OH^-^ ions into the system [12,13]. On the other hand, the alkalinization of the aqueous medium also promotes the dissolution of SiO_2_, generating metasilicic acid (H_2_SiO_3_) and orthosilicic acid (H_4_SiO_4_). Under these conditions of the aqueous medium, the reaction between MgO/Mg(OH)_2_ and silicic acid species results in the precipitation of a hydrous magnesium silicate with a low degree of crystallinity, commonly referred to in the literature as M-S-H gel [14]. As mentioned, in these systems, alongside M-S-H gel formation, another hydration reaction occurs, leading to the precipitation of brucite [Mg(OH)_2_] crystals. Despite its immediacy, the formation of Mg(OH)_2_ has not received equal attention due to potential expansive issues in conventional systems [15]. Furthermore, Mg(OH)_2_ crystals lack the binding properties demonstrated by M-S-H gels, explaining the limited interest in literature.

Despite these advantages, the application of MgO-SiO_2_ systems is limited by the slow kinetics of M-S-H gel formation, which hampers the overall efficiency of the system. While various approaches have been explored to accelerate M-S-H formation, such as optimizing the mixing process or incorporating additives, these methods often come with their own limitations or added costs. The formation of M-S-H is enhanced by the addition of hydromagnesite [16]. However, this additive is a rare hydrated magnesium carbonate that is difficult to obtain directly from mining processes and requires synthesis for its production [17]. On the other hand, the addition of alkali carbonates [18,19] also significantly improves the hydration degree of these systems but adversely affects their workability, requiring a higher amount of mixing water.

Moreover, the high cost of amorphous SiO_2_, which is essential for optimal M-S-H production, further restricts its use. In this context, exploring high MgO content M-S-H systems offers a promising alternative. By utilizing MgO sourced from by-products, such as desalination brine or magnesium silicate ore waste, this approach aims to reduce reliance on expensive amorphous SiO_2_, making M-S-H cement production more cost-effective. Furthermore, natural pozzolans could be used in synergy to further reduce costs [20]. Additionally, carbonation presents a crucial technique to enhance these high MgO content systems. Exposing the cementitious pastes to a CO_2_-saturated atmosphere facilitates the conversion of Mg(OH)_2_ crystals into hydrated magnesium hydroxycarbonates. This carbonation process, represented by the equation (Equation (1)), not only refines porosity and improves bonding properties but also provides a method for encapsulating CO_2_ in Mg-based cement precast products:

(1) $$x\;{Mg(OH)}_{2}+y\;H_{2}O+zCO_{2}\to \alpha MgCO_{3}\cdot \beta \left(OH\right)\cdot \gamma H_{2}O$$

This study investigates the impact of early exposure to a CO_2_-saturated atmosphere—conducted at 4 and 25 days of curing for 72 h—on the mechanical strength development and microstructural changes in MgO-SiO_2_ cementitious systems during complete curing periods of 7 and 28 days. By investigating the impact of early carbonation on the properties of M-S-H systems, this study seeks to lay the groundwork for viable, high-MgO content M-S-H cements. It aims to propose alternatives and solutions to enhance economic feasibility and support sustainable CO_2_ encapsulation in Mg-based precast cement products.

## 2. Results and Discussion

### 2.1. Compressive Strength

**Figure 1:**
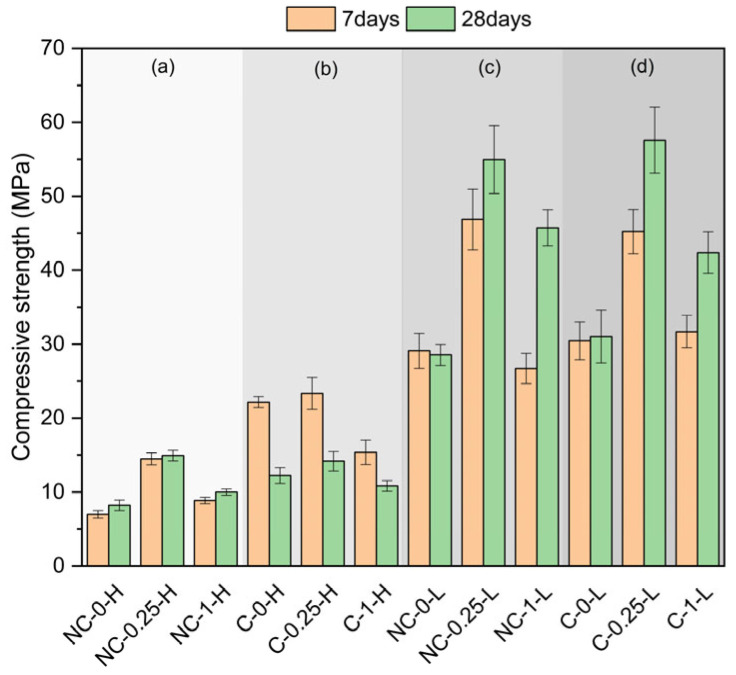
Compressive strength of the different formulations studied as a function of the water-to-cement ratio.

Figure 1 displays the compressive strength of the assessed samples. At 7 days, NC-0.25-H (20% SiO_2_) exhibits the highest compressive strength in section (a), followed by NC-1-H (50% SiO_2_), with NC-0-H (no SiO_2_) showing the lowest strength. The addition of 20 wt% SiO_2_ results in a significant strength increase compared to samples with no SiO_2_ content. In section (b), which includes carbonated samples with high water demand, C-0.25-H (20% SiO_2_) has the highest compressive strength, similar to C-0-H (SiO_2_-free); C-1-H (50% SiO_2_) shows the lowest strength. Carbonation and low SiO_2_ content lead to a notable increase in compressive strength.

In section (c), involving non-carbonated samples with low water demand, NC-0.25-L (20% SiO_2_) demonstrates the highest compressive strength at 7 days, followed by NC-0-L (no SiO_2_), with NC-1-L (50% SiO_2_) showing the lowest strength. The lower water demand significantly improves the strength for all samples compared to those in section (a). In section (d), which includes carbonated samples with low water demand, C-0.25-L (20% SiO_2_) shows the highest strength, while C-1-L (50% SiO_2_) and C-0-L (no SiO_2_) exhibit similar compressive strengths. Comparing sections (c) and (d), C-0-L and C-1-L have higher compressive strengths compared to non-carbonated samples in section (c), emphasizing the effect of carbonation combined with lower water demand. Overall, at 7 days, a 20% SiO_2_ content consistently improves compressive strength. Both carbonation and lower water demand significantly enhance compressive strength, especially when combined. The most effective combination for early strength development is observed in samples with 20% SiO_2_ and low water demand. The most significant difference between different ages is that compressive strength at 28 days is generally higher than at 7 days for most samples, which is typical in cementitious materials due to continued hydration processes. However, the extent of strength gain from 7 to 28 days varies across samples, and in some cases, the opposite occurs. For instance, carbonated samples at 28 days with high water demand exhibit decreased mechanical strength compared to the same formulations carbonated at 7 days. In sections (c) and (d), which involve samples with low water demand, the 28-day strength values are significantly higher than the 7-day values, particularly for NC-0.25-L and C-0.25-L, suggesting that low water demand samples benefit more from extended curing times.

### 2.2. Hg-Intrusion Porosimetry

**Figure 2:**
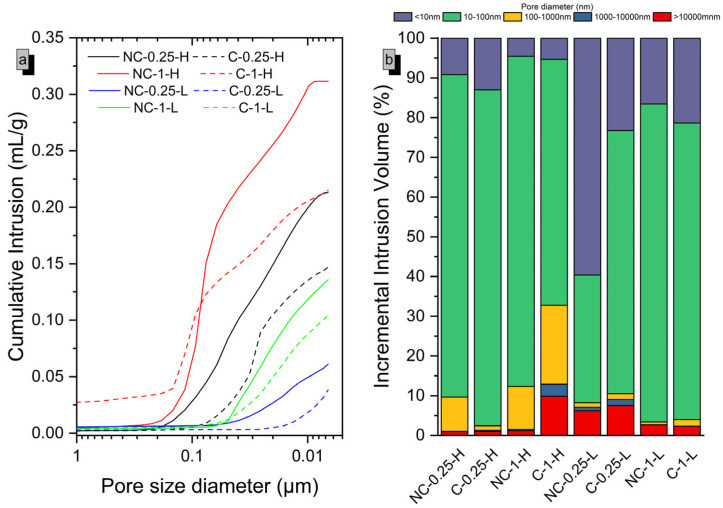
Mercury intrusion cumulative curves, cumulative intrusion (**a**) and incremental intrusion volume (**b**), as a function of pore size for the various MgO-SiO_2_ systems analyzed in this study at 7 days of age.

The results from Figure 2 indicate that non-carbonated samples with 50 wt% SiO_2_ content (NC-1) exhibit greater porosity compared to those with lower SiO_2_ content (NC-0.25), regardless of the water-to-cement ratio (0.8 or 0.42). This can be attributed to the high surface area of the silica fume used, which adsorbs more water, leading to less efficient compaction during mixing. This increased porosity in NC-1 suggests less efficient compaction and more void spaces, which would likely reduce the compressive strength. In contrast, the carbonation process helps to reduce this porosity, leading to improved mechanical performance. The reduced porosity in carbonated samples aligns with the observed increase in strength.

When comparing samples with different water-to-cement ratios, those with higher ratios exhibit greater porosity due to the excess water in the mix, leading to additional voids once the samples are hydrated. This higher porosity results in weaker compaction, which negatively impacts mechanical strength in non-carbonated samples, as observed in the previous mechanical results. Upon subjecting the samples to a CO_2_-saturated atmosphere, a reduction in porosity is observed compared to the non-carbonated samples, regardless of the water-to-cement ratio. The reduction is particularly notable in the refined pore range, depending on the ratio employed. In Figure 2, samples with a lower ratio (0.42) show only a slight reduction in porosity for pores smaller than 0.05 μm, likely occurring within particle agglomerate pores [1,21]. On the other hand, samples with a higher water-to-cement ratio also experience a clear reduction in capillary porosity [21], with reductions in cumulative intrusion values of 30.2% and 70.5% for samples with SiO_2_ contents in their composition of 50 wt% and 20 wt%, respectively. This reduction in porosity is linked to the improved compressive strength observed in the carbonated samples, which justifies the significant strength gain exhibited by the samples with higher water-to-cement ratios at early ages. However, the refinement benefit induced by the excess porosity associated with a high water-to-cement ratio may not be sufficient to offset the loss in mechanical strength resulting from this excessive porosity. It is likely that this elevated water-to-cement ratio (0.8) requires optimization to enhance the overall mechanical performance. These findings suggest that porosity significantly influences the carbonation of the analyzed systems. The excess porosity in the matrix serves two primary purposes. Firstly, it facilitates the diffusion of CO_2_ (gas) throughout the matrix, ensuring it reaches all regions uniformly, and secondly, it allows the presence of essential water for the dissolution of Mg(OH)_2_ and CO_2_, leading to the formation of carbonic acid. Based on porosimetry results, it is hypothesized that exposure to a CO_2_-saturated atmosphere induces significant changes in the microstructure and phase composition of MgO-SiO_2_ systems. The observed increase in solid volume and porosity refinement are proposed to hinder the diffusion of CO_2_ and the dissolution of reactants. This hypothesis will be further examined in subsequent sections using complementary characterization techniques such as XRD, TGA, FTIR, SEM, and EDS. Thus, an initial excess of porosity is crucial for achieving a high degree of carbonation.

### 2.3. XRD

**Figure 3:**
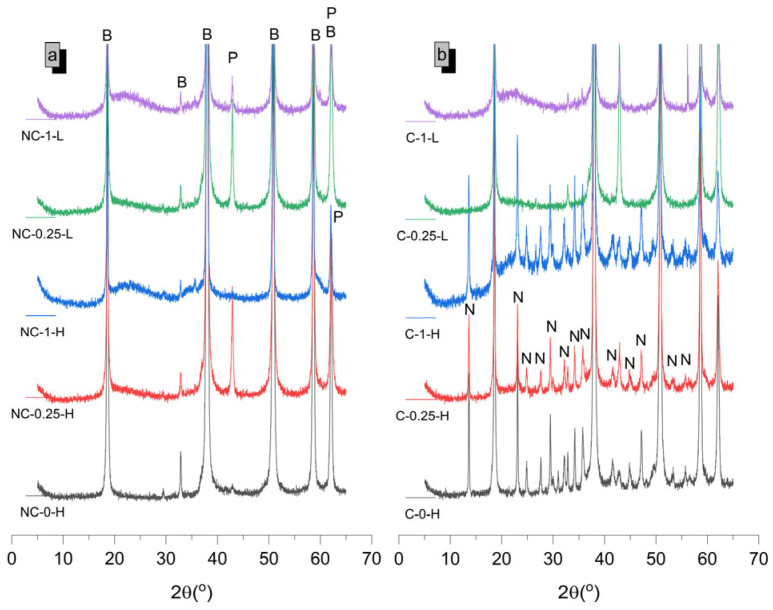
Diffractograms of samples, both non-carbonated (**a**) and carbonated (**b**), at 7 days of age. B, P and N refer to brucite, periclase and nesquehonite respectively.

**Figure 4:**
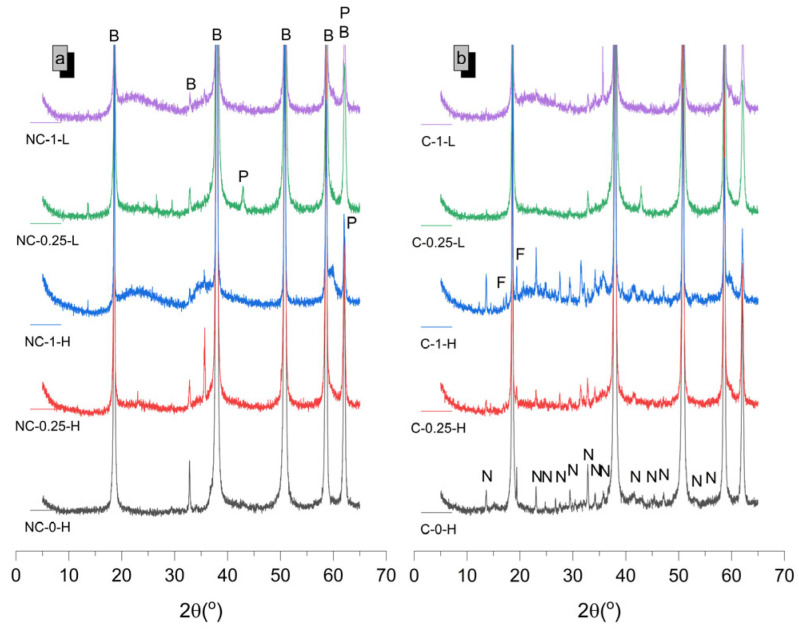
Diffractograms of samples, both carbonated (**a**) and non-carbonated (**b**), at 28 days of age. B, P, N, and F refer to brucite, periclase, nesquehonite, and lansfordite, respectively.

**Figure 5:**
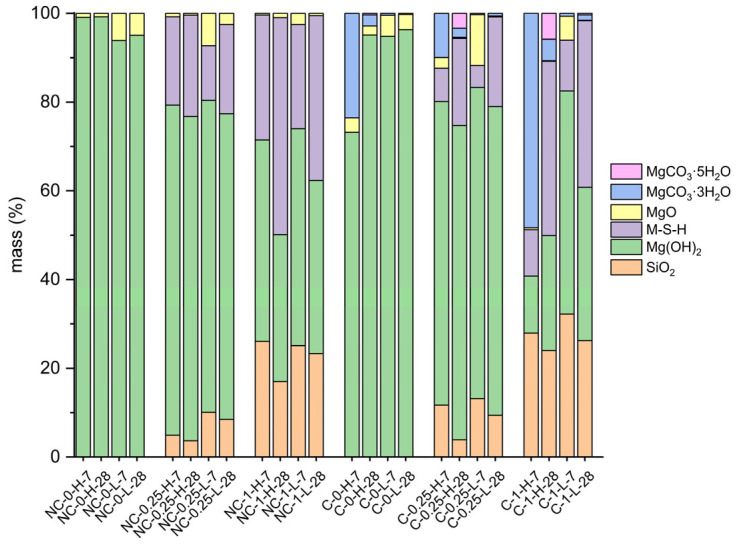
Mass fraction (wt%) of each of the samples analyzed through phase quantification via Rietveld refinement.

The diffractograms for samples at 7 and 28 days (Figure 3 and Figure 4) are shown below, with phase quantifications in Figure 5. The complete graphs, showing each diffractogram with peaks at their maximum intensity, are provided in Figures S1 and S2 (Supplementary Materials).

In 100% MgO non-carbonated (NC) samples, the absence of SiO_2_ limits hydration to Mg(OH)_2_ (COD-ID: 2101438) formation. Higher water-to-cement ratios increase Mg(OH)_2_ formation due to more available water. XRD results (Figure 5) indicate incomplete hydration, with unreacted MgO content of 6.12 wt% at 7 days and 4.96 wt% at 28 days. This may be due to Mg(OH)_2_ precipitating on MgO (COD-ID: 4111968) particles, potentially inhibiting further dissolution, as suggested by prior studies [13,22]. When these 100% MgO systems are carbonated (C) in high humidity, HMHCs with the general formula αMgCO_3_·β(OH)·γH_2_O form [23,24]. Higher water-to-cement ratios promote a higher carbonation degree (Figure 5) by increasing initial porosity, which enhances CO_2_ diffusion. Figure 5 also shows that HMHC formation is primarily due to the carbonation of Mg(OH)_2_, with unreacted MgO content remaining stable. Increased carbonation in early-age samples (7 days), particularly those with a water-to-cement ratio of 0.8, may be attributed to the lower stability of Mg(OH)_2_ compared to 28 days. This increased porosity, coupled with carbonation, justifies the significant strength gain observed in these samples at early ages. At 7 days, nesquehonite (MgCO_3_·3H_2_O, COD-ID: 9012401) is the main carbonate phase, while lansfordite (MgCO_3_·5H_2_O, COD-ID: 9012797) appears at 28 days. Converting Mg(OH)_2_, and to a lesser extent unreacted MgO, into MgCO_3_·3H_2_O requires several intermediate steps as shown in the following equation (Equation (2)):

(2) $${Mg(OH)\;}_{2}+{CO}_{2}+{2H}_{2}O\;\to \;{Mg(OH)}_{2(aq.)}+H_{2}{CO}_{3}+H_{2}O\to \;{CO}_{3}^{2-}+{Mg}^{2+}+{3H}_{2}O$$

The late formation of nesquehonite and lansfordite suggests an equilibrium between water availability and carbonation conditions. The interaction between phases and the material′s microstructure allows for the concurrent formation of both phases, despite lansfordite being thermodynamically less stable [25,26].

In NC SiO_2_-containing samples, Mg(OH)_2_ and M-S-H gel form. Higher SiO_2_ content (50 wt%) promotes more M-S-H gel formation, consistent with previous research [9,27]. NC-0.25-L samples retain more unhydrated MgO and significant Mg(OH)_2_ content at 7 days, similar to 100% MgO systems. Conversely, higher water-to-cement ratio samples show more M-S-H gel and less MgO, in line with earlier findings [8]. By 28 days, increased M-S-H gel formation corresponds with reduced Mg(OH)_2_ content [9,12,13].

Additionally, carbonated samples with 50 wt% SiO_2_ content (C-1-H and NC-1-L) show more carbonation compared to 20 wt% SiO_2_ content samples (C-0.25-H and NC-0.25-L) due to their higher porosity, which facilitates CO_2_ penetration, even though they contain lower MgO content susceptible to carbonation. Moreover, samples containing SiO_2_ with a water-to-cement ratio of 0.42 exhibit carbonation trends similar to those of 100% MgO samples with a low water-to-cement ratio, as both systems show a lower proportion of HMHC, regardless of CO_2_ exposure age. For these water-to-cement ratios, the HMHC content is lower than 1 wt% of the composition. This aligns with the principle observed in SiO_2_-free samples, indicating notable stability against carbonation under the studied curing conditions (CO_2_-saturated atmosphere for 72 h). Carbonation in these samples may be limited to the surface, potentially leaving the interior unaffected by CO_2_-related effects. Additionally, HMHC formation decreases with age, likely due to the greater stability of Mg(OH)_2_ at later ages and a more refined microstructure, both of which hinder carbonation over time. To mitigate carbonation, controlling initial curing conditions, even with high water-to-cement ratios, is crucial. Similarly, both SiO_2_-containing samples exhibit only MgCO_3_·3H_2_O formation at early ages, transitioning to an additional phase, MgCO_3_·5H_2_O, at advanced ages. A key observation is that HMHC formation is not only tied to a decrease in Mg(OH)_2_ content when precipitating >1 wt% of HMHC. It is also associated with a reduction in M-S-H gel content in systems with significant HMHC precipitation (>1 wt%). The reduction in M-S-H gel content can be attributed to the saturation of the system with CO_2_ during carbonation. In a CO_2_-rich environment, the carbonation reaction competes with the formation of M-S-H gel, leading to a decreased rate of gel formation. This suggests that the presence of high CO_2_ concentrations hinders the reaction responsible for M-S-H gel formation, as CO_2_ likely disrupts the equilibrium or the availability of reactive species necessary for the gel′s development.

### 2.4. TG Analysis

**Figure 6:**
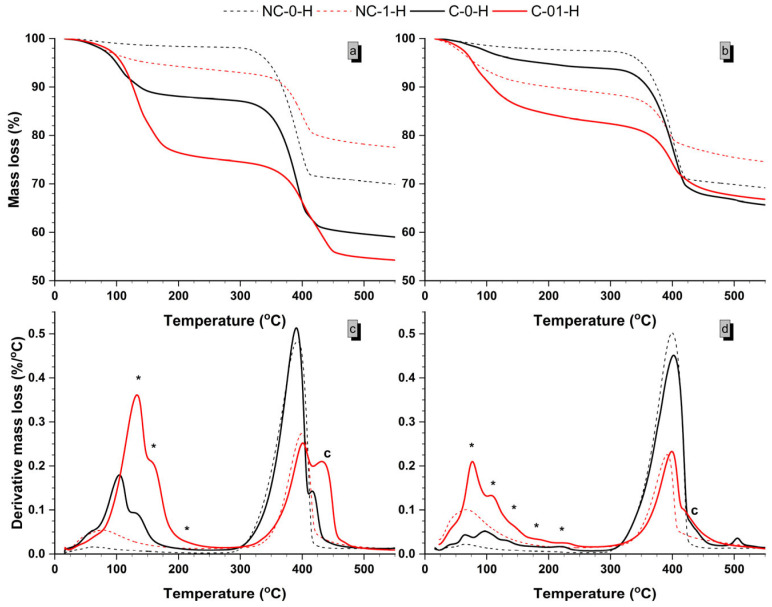
Representation of mass loss versus temperature increase in the selected samples (NC-0-H, NC-1-H, C-0-H and C-1-H) at 7 days (**a**) and 28 days (**b**). Representation of the rate of mass loss versus temperature increase in the samples at 7 days (**c**) and 28 days (**d**). The asterisk symbols (*) indicate mass losses due to the presence of products not observed in NC samples.

All samples underwent TG/DTG analysis, revealing two major stages of mass reduction: up to 250 °C (P250) and up to 550 °C (P550), corresponding to the decomposition of components in the hydrated matrix influenced by formulation and CO_2_ exposure. Figure 6 illustrates the DT/DTG curves for non-carbonated (NC) and carbonated (C) samples with a w/b ratio of 0.8, highlighting the effects of carbonation. In Figure 6a,b, mass loss increases with temperature, while Figure 6c,d display the rate of mass loss at 7 and 28 days for samples with a w/b ratio of 0.8. The figures compare samples with maximum (NC-0-H and C-0-H, black lines) and minimum (NC-1-H and C-1-H, red lines) MgO content.

**Figure 7:**
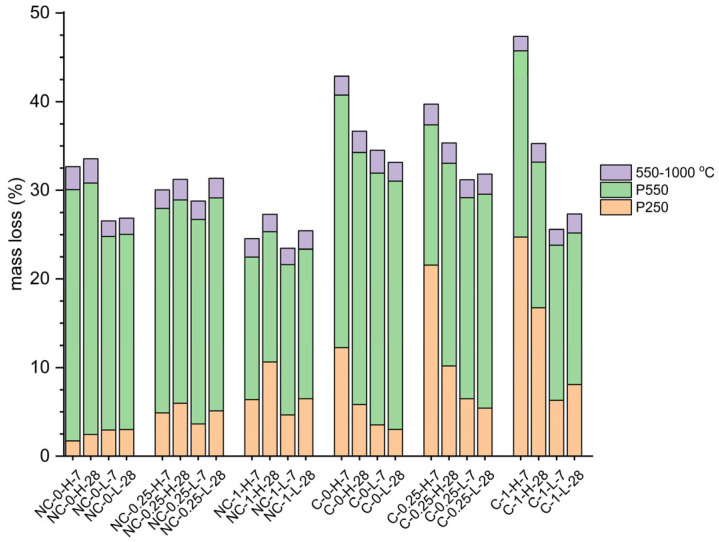
Mass loss values across temperature ranges for analyzed formulations considering composition, CO_2_ exposure, and age.

In NC samples, P250 values (Figure 7) are attributed to the loss of adsorbed moisture. For these samples, P550 values from Mg(OH)_2_ dehydroxylation are 28.34% and 28.37% at 7 and 28 days, respectively (Figure 7). This mass loss estimates Mg(OH)_2_ content at 92.00% and 91.81%, slightly lower than XRD values. For NC-1-H samples, P250 values are higher: 6.38% at 7 days and 10.62% at 28 days (Figure 7). This increase is due to both the loss of adsorbed moisture and interlamellar water loss from the M-S-H gel, indicating a greater presence of M-S-H gel over time for these formulations [1,12]. However, in NC-1-H samples, P550 values cannot be solely attributed to Mg(OH)_2_ dehydroxylation. Although the thermal decomposition of Mg(OH)_2_ is a major contributor to mass loss in this temperature range, it is not the only factor. The M-S-H gel also loses mass due to the dehydroxylation of (OH)^-^ groups, leading to overlapping dehydroxylation processes of both Mg(OH)_2_ and M-S-H [1].

In C-0-H and C-1-H, the same P250 and P550 temperature ranges for major mass losses are observed as in NC samples, but with higher mass losses (Figure 7). The derivative mass loss curves C-0-H and C-1-H also show larger endothermic peaks with multiple shoulders in the P250 range, in contrast to the single broad peak in NC samples. This additional mass loss, linked to water release from various HMHC as marked by asterisks, indicates the presence of products not seen in NC samples [28,29]. At 7 days, decomposition in the P250 range includes water loss from both the M-S-H gel and MgCO_3_·3H_2_O, which typically loses water at 55–67 °C and 240–254 °C [28]. Figure 6c shows two distinct endothermic peaks, while the third peak around 220 °C is barely noticeable. For C-1-H samples, the main peak is prominent around 133 °C with a shoulder at 160 °C. In contrast, C-0-H samples show peaks at 130 °C and 100 °C, respectively. This indicates that the addition of SiO_2_ results in more thermally stable phases. In the P550 range, carbonated samples show a shoulder around 410–440 °C, related to the decarbonation of MgCO_3_·3H_2_O [28,29,30]. The CO_2_ loss from this carbonate begins around 300–350 °C, with peak decomposition from 400 °C onwards [28,30]. This decarbonation peak occurs at higher temperatures in C-1-H samples.

At 28 days, the P250 temperature range shows more shoulders on the endotherms than at 7 days, revealing up to five peaks. Two peaks below 100 °C likely correspond to the dehydration of MgCO_3_·5H_2_O to MgCO_3_·3H_2_O [25]. Above 100 °C, water loss is due to the dehydration of MgCO_3_·3H_2_O, from nesquehonite formed during carbonation and trihydrate from lansfordite decomposition. A shoulder around 400 °C, associated with decarbonation, appears similarly to the 7-day carbonated samples. At 28 days, this decarbonation peak overlaps with the dehydroxylation of the M-S-H gel, which has increased in quantity. Additionally, a subtle endothermic peak around 500 °C is observed, linked to the decarbonation of lansfordite [25]. Mass losses observed above 550 °C in M-S-H cement are considerably smaller than those at lower temperatures. These losses are primarily due to the dehydration of magnesium hydroxide and the partial structural breakdown of the M-S-H gel. [1,12] Additionally, beyond this temperature range, no significant influence of carbonate presence is observed, indicating that they are not involved in the thermal processes within this temperature interval.

**Figure 8:**
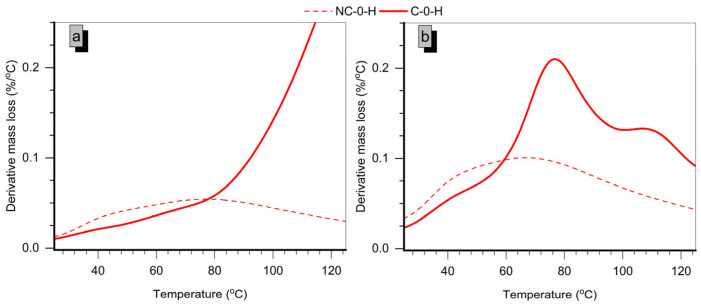
Expansion of the thermogravimetric derivative curves of the 50% MgO-50% SiO_2_ samples up to 125 °C at (**a**) 7 days and (**b**) 28 days (dashed lines represent curves of non-carbonated samples, while solid lines correspond to curves of carbonated samples).

Lastly, C-0-H samples show a reduction in the endothermic curve for interlaminar water loss from the M-S-H gel (dashed line in Figure 8) at both ages, despite higher P250 values. This reduction suggests a lower Mg content in the M-S-H gel, consistent with XRD findings that show not only reduced Mg(OH)_2_ but also a decrease in M-S-H gel. The lower Mg/Si ratio in these gels could be linked to the carbonation of the M-S-H gel, which likely leads to the loss of Mg.

### 2.5. FTIR Analysis

**Figure 9:**
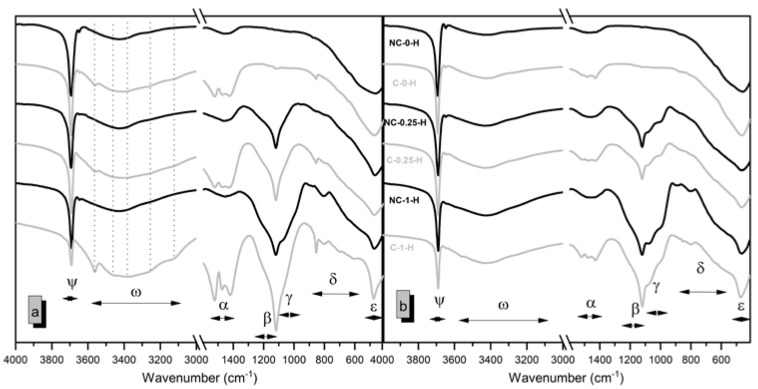
FTIR spectra of selected samples at 7 (**a**) and 28 (**b**) days. The Greek letters correspond to the different bands discussed in the text.

The evolution of hydration reactions over time and their correlation with potential exposure to a CO_2_-saturated environment can be analyzed from Figure 9, considering the formed bonds.

In samples not exposed to a CO_2_-rich environment (indicated by the black lines), primary hydration reactions involve Mg(OH)_2_ and M-S-H gel precipitation, consistent with literature findings [12,13] and previous sections. The precipitation of brucite is evident in all samples (peak Ψ in Figure 9), with a prominent peak around 3690 cm^−1^ and a minor replica at 3650 cm^−1^, attributed to the O-H stretching vibration in Mg(OH)_2_ [1]. The intensity of this peak remains consistent across both studied ages (7 and 28 days), being higher in samples with higher MgO content. A broad band between 3050 and 3600 cm^−1^ (ω in Figure 9) is attributed to the O-H stretching band of water [31] due to chemically adsorbed water by the samples during handling, and is consistent across all formulations at 7 days.

At 28 days, samples with SiO_2_ show increased intensity in band ω, indicating more OH groups in the M-S-H gel [1,32]. This aligns with observations in TG curves. Similarly, a weak band between 1400 and 1600 cm^−1^ (band α), attributed to the hydroxyl stretch [14], also intensifies in SiO_2_-containing samples at 28 days, indicating a higher content of OH groups in the M-S-H gel. SiO_2_ reactivity is evident in the 900–1300 cm^−1^ range (bands β and γ). The signals in the range of 1100–1250 cm^−1^ (band β) correspond to Si-O vibration (Q3) [7] and coincide with the prominent band in the spectra of unreacted SF (SiO_2_) as well as the band at 810cm^−1^ associated with the lattice stretching of SiO_2_ [14]. The β band is absent in the 100% MgO samples (NC-0-H) and intensifies with the increase in SiO_2_ content in the sample formulation. Signals in band γ, with shoulders at 1000 and 1070 cm^−1^, correspond to Q2 Si–O stretching in M-S-H gels [7] and are more pronounced in samples with higher SiO_2_ content, particularly at advanced ages. Another peak at 890 cm^−1^ corresponds to Si–O vibration (Q2), indicative of M-S-H gel formation [7].

The effects of carbonation are evident in the bands between 3050–3650 cm^−1^ (ω), 1300–1600 cm^−1^ (α), and 550–875 cm^−1^ (δ). In the ω bands, associated with free and chemically adsorbed water in non-carbonated samples, carbonated samples show that weak peaks at 3115, 3250, 3340, 3460, and 3560 cm^−1^ appear in carbonated samples, corresponding to nesquehonite presence. These peaks represent water and OH stretching vibrations in nesquehonite [33,34]. In the α band (1300–1600 cm^−1^), peaks absent in non-carbonated samples, such as those at 1420 and 1470 cm^−1^ and at 1523 cm^−1^, indicate CO_3_^2−^ and HCO_3_^−^ antisymmetric stretching modes, respectively. The band δ shows peaks at 860 cm-1 (out-of-plane ν2 CO_3_^2−^ and ν2 HCO_3_^−^ bending), 713 cm^−1^ (in-plane bending mode (ν4) CO_3_^2−^), and 610 cm^−1^ (ν4 in-plane bending mode of the HCO_3_^−^ units). These signals demonstrate higher intensity in carbonated samples at 7 days. Additionally, carbonation leads to a reduction in intensity of the γ region shoulders, associated with Q2 Si–O stretching vibration in the M-S-H gels of non-carbonated samples, indicating a lower amount of gel in carbonated samples, both at 7 and 28 days.

### 2.6. SEM-EDS

**Figure 10:**
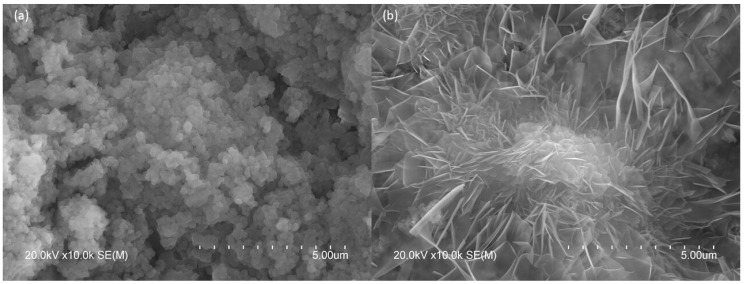
Images of the different particle morphologies, (**a**) non-carbonated compounds and (**b**) carbonated compounds, observed in the samples (C-1-H at 7 days) subjected to a CO_2_-saturated atmosphere.

The SEM images show the main crystalline phases present in the studied systems after subjecting them to a CO_2_-saturated environment: brucite (Figure 10a) and HMHC hydroxyl-hydrate magnesium carbonates (Figure 10b). Brucite appears as uniform-sized platelets < 1 µm, forming larger and compact clusters, consistent with previous studies on MgO hydration [35,36,37,38]. The hydroxyl-hydrate magnesium carbonates exhibit rosette-shaped particles resembling hydromagnesite [39,40,41]. The presence of these characteristic hydromagnesite particles contrasts with the results obtained by XRD, where no crystalline pattern of these compounds was observed in any case. Furthermore, the presence of hydromagnesite could also be ruled out based on the results of TG and FTIR, where no coincident findings were observed in any case, based on studies examining MgO-hydromagnesite mixtures [42].

The presence of nesquehonite crystals exhibiting a morphology different from that reported in most studies can be justified by a confinement process within spatial networks. In this context, nesquehonite can take on a rosette-like shape, as shown in other studies where specific additives—such as dextran, MgCl_2_·6H_2_O with NH_3_·H_2_O, or magnesium salts combined with carbonate solutions—induce the growth of porous spherical and rose-like structures. Under these conditions, nesquehonite sheets grow confined within a spatial network, which restricts solute diffusion and promotes distance nucleation between the nanosheets. This setup encourages the formation of porous spheres through epitaxial growth, where the rotation of the nanosheets produces a rosette-like structure [43,44,45].

EDS measurements (Table 1) of the Mg/Si ratio were conducted on samples with 50 wt% SiO_2_ content due to easier area selection, minimizing interference from other particles like brucite. This was challenging in samples with 20% SiO_2_, where isolating areas containing only M-S-H gel was difficult. Analysis focused on 7-day samples with a water-to-cement ratio of 0.8, considering increased carbonation. Results show similar Mg/Si values (1.18 to 1.35) across most samples, with a notable decrease in the carbonated sample (C-1-H) compared to non-carbonated (NC-1-H). This reduction suggests a lower Mg content in the M-S-H gel, consistent with prior findings that show not only reduced Mg(OH)_2_ but also a decrease in M-S-H gel. The lower Mg/Si ratio in these gels could be linked to the partial carbonation of M-S-H gel, which likely leads to the loss of Mg. In samples with lower water-to-cement ratios (0.42), a slight increase in Mg/Si ratio and unreacted SiO_2_/MgO values implies gel depletion without additional carbonate formation in CO_2_-rich atmospheres.

## 3. Materials and Methods

### 3.1. Materials

The raw materials used for sample preparation consist of magnesium oxide (MagChem 30, Martin Marietta Materials, Raleigh, NC, USA) and silica fume (Elkem Microsilica Grade 955, Elkem, Oslo, Norway). Table 2 shows the main characteristics of the raw materials employed in this study.

Paste samples were prepared following the compositions listed in Table 3. To ensure comparable workability across formulations, different superplasticizer contents (Sika Viscocrete-520P (Sika Germany, Stuttgart, Germany)) were used, along with varying water-to-cement ratios (0.42 and 0.8), to achieve distinct porosity levels in the hardened pastes. Workability was controlled using a mini-slump cone test, with the superplasticizer dosage adjusted according to the SiO_2_/MgO ratio. Specifically, 0.8%, 0.5%, and 0.35% of the superplasticizer (by total cement mass) were used for SiO_2_/MgO ratios of 0, 0.25, and 1, respectively.

### 3.2. Sample Preparation and Curing

Paste samples were prepared as follows: Initially, dry MgO and SiO_2_ powders were blended in a high-shear mixer at 180 rpm for 5 min. Subsequently, water was added, and all components were mixed at 1600 rpm for 5 min. The resulting paste was then poured into 1 × 1 × 6 cm^3^ prismatic steel containers and cured under controlled humidity conditions at 99% relative humidity and 22 °C ± 2 °C until the designated testing or carbonation ages according to [46]. Reference samples (non-carbonated, labelled as NC- in Table 3) were cured in a climatic chamber at the controlled humidity room until reaching the selected analysis ages (7 and 28 days). Carbonated samples were exposed to a saturated CO_2_ (CO_2_ ≥ 99.9 Vol.%) atmosphere (labelled as C- in Table 3) at 70% relative humidity and 20 °C in an airtight container connected to a pressurized CO_2_ bottle. To establish CO_2_ saturation, three cycles of vacuum (0.2 bar for 10 min) followed by CO_2_ injection (1 atm for 5 min) were performed. After these cycles, the samples were maintained at 1 atm CO_2_ pressure for 3 days before analysis. CO_2_ curing started at day 4 for 7 days samples and at day 25 for 28 days samples.

### 3.3. Methods

Compressive strength was assessed after 7 and 28 days, with six replicates per series tested using a universal testing machine Ibertest-Autotest 300/20 (Madrid, Spain) equipped with a 20 kN load cell at a speed of 0.07 kN/s. The mechanical results were processed as detailed in Appendix A. X-ray diffraction (XRD), thermogravimetric analysis (TG), and Fourier Transformed Infrared Spectroscopy (FTIR) were performed once sample hydration was halted after their mechanical characterization. The samples, initially milled finer than 63 μm, were mixed with excess isopropyl alcohol in a glass beaker, stirred for 2 min, filtered under vacuum, dried, transferred to a watch glass, vacuumed in a desiccator for 2 min, stored for 48 h, and finally stored in a dry place.

X-ray diffraction (XRD) was employed for the identification and quantification of phases present in the pastes at different hydration ages analyzed: 7 and 28 days. X-ray diffraction testing was conducted using a D8 ADVANCE instrument (^®^Bruker AXS (Billerica, MA, USA)), equipped with a 2.5° Soller slit, an ultra-fast RX “Lynxeye” detector (Billerica, MA, USA) (including a 3 mm anti-dispersion slit, a 2.5° Soller slit, and a 0.5% Ni K-beta filter). The test is performed without a monochromator using Cu Kα1 = 1.54056 nm and Cu Kα2 = 1.54439 nm wavelengths. The parameters include: goniometer radius: 217.5 mm, voltage generator: 40 kV, current generator: 30 mA, fixed divergence slit: 0.5°, time per step: 5 s, step: 0.01973°, and testing range: 2θ = 5–65°. For phase identification, DIFFRAC.EVA v.4.2.1.10 software (32 bits) was used, while for Rietveld refinement and the quantification of these phases, TOPAS v.5 software, both owned by ^®^Bruker AXS (Billerica, MA, USA), were employed.

For phase quantification, a PONKCS (Partial Or No Known Crystal Structures) technique was employed due to the presence of two types of components with little to no known crystalline structure prior to Rietveld refinement. The procedure follows the method described in previous works [47,48]. This approach enables the calibration of the ZMV constant for a material of unknown structural information. To execute the ZMV calibration, the procedure is as follows:

1. The diffractogram of the amorphous phases to be calibrated is collected (in this case, silica fume and synthetically precipitated M-S-H gel). M-S-H gel with an approximate Mg/Si ratio of 1.2 was targeted, as it resembles the ratio of the gel detected by EDX and is precipitated following the method described by [14].
2. Each diffractogram is fitted to an hkl-phase adjusted with Topas Software (Version 5) to determine all planes (hkl), initially assigning P4_1_2_1_2 and P1m1 space groups to silica fume and M-S-H gel, respectively. These space groups correspond to cristobalite (SiO_2_) and antigorite [Mg_3_Si_2_O_5_(OH)_4_], identified by COD-IDs 1010938 and 9004514, respectively. Both are crystalline materials with a chemical composition similar to that of the phases with an unknown structure. Once the diffractogram is adjusted, the hkl planes and all lattice parameters are fixed, and the cell volume (V) is calculated for each adjusted hkl phase.
3. The ZMV (molar volume constant) of each amorphous phase is calibrated using a mixture with a known quantity of the unknown amorphous phase (α) and a reference material (s). This allows the determination of the cell mass (M) for our amorphous phases using the following equation (Equation (3)):
  (3) $${\left(ZM\right)}_{\alpha }=\frac{W_{\alpha }}{W_{s}}\cdot \frac{S_{s}}{S_{\alpha }}\cdot \frac{{\left(ZMV\right)}_{s}}{V_{\alpha }}$$
  where Z is the number of units per cell, S is the scale factor, and W is the mass fraction for each phase.

Thermogravimetric analysis (TG) was performed using an SDT-Q600 instrument (^®^TA Instruments (New Castle, DE, USA)), placing the samples in an Al_2_O_3_ crucible and heating them from 10 °C to 1000 °C at a rate of 10 °C/min in a nitrogen atmosphere. For Fourier Transform Infrared (FTIR) analysis, a Nicolet 5700 spectrometer (Waltham, MA, USA) as utilized in the range of 400 cm^−1^ to 4000 cm^−1^ at a resolution of 4 cm^−1^ using powder samples (<63 μm). The powdered material was mixed with KBr granules (0.001 g of sample/0.099 g of KBr) and subsequently formed into pellets through mechanical pressing. For Hg porosimetry intrusion and SEM-EDS characterization, sections of solidified paste specimens at the specified age were submerged in isopropanol for 48 h to halt reactions, and subsequently placed in a desiccator for a minimum of 48 h to eliminate any remaining isopropanol. Alterations in the pore configuration were assessed using MIP. The MIP was performed on a Micrometrics AutoPore IV 9500 mercury intrusion porosimeter (Norcross, GA, USA), which operates at a maximum pressure of approximately 2276 bar, assuming a sample mercury contact angle of 141.3°. The microstructure of the specimens was examined utilizing a Hitachi S-4800 scanning electron microscope (Tokyo, Japan). Samples were embedded in epoxy resin, sanded with progressively finer sandpapers, polished with diamond pastes, treated with ultrasonic cleaning, air-dried, coated with carbon, and inspected under high vacuum conditions (20 kV). Furthermore, a semiquantitative analysis of the Mg/Si of the M-S-H gel was conducted using EDX spectroscopy on a Bruker Flash Detector 5030 EDX analyzer (Billerica, MA, USA). This procedure entailed collecting a minimum of 30 representative data scanning from the M-S-H gel area per sample, followed by data processing using the Bruker ESPRIT 2 software (Billerica, MA, USA).

## 4. Conclusions

This study has demonstrated that early exposure to a CO_2_-saturated atmosphere significantly impacts the hydration kinetics and mineralogical development of MgO-SiO_2_ cementitious systems. The results highlight several key conclusions:

Carbonation behavior is significantly influenced by the SiO_2_/MgO ratio, water-to-cement ratio, and the age at which carbonation occurs. Higher SiO_2_ content increases initial porosity, facilitating greater CO_2_ diffusion and enhancing carbonation, even with reduced MgO availability. However, the accompanying porosity compromises compaction, limiting mechanical performance gains. In contrast, a lower water-to-cement ratio reduces initial porosity, resulting in less carbonation but improved strength due to denser microstructures. The balance between carbonation-induced densification and the destabilization of reactive phases, such as Mg(OH)_2_, plays a critical role in determining the mechanical performance at both early and later ages.

Carbonation leads to the formation of hydrated carbonates, primarily nesquehonite (MgCO_3_·3H_2_O) and lansfordite (MgCO_3_·5H_2_O), with the proportions influenced by water availability and system stability. At early ages, increased carbonation due to the lower stability of Mg(OH)_2_ and higher porosity enhances compressive strength. However, at later ages, the carbonation reaction slows as the microstructure becomes more refined, reducing the rate of CO_2_ penetration. This interplay highlights the pivotal role of porosity not only in enabling carbonation but also in defining the evolution of mechanical properties and phase stability over time.

These findings suggest that managing early CO_2_ exposure can effectively enhance the mechanical properties of M-S-H binders with high MgO content. Future research should focus on exploring the long-term stability of nesquehonite and lansfordite, as well as optimizing mix designs to reduce reliance on costly reactive SiO_2_, thereby improving the cost-effectiveness and performance of MgO-SiO_2_ cementitious systems.

## Figures and Tables

**Table 1:** The Mg/Si atomic ratio of the samples with high SiO_2_ content in their composition obtained by EDS at 7 days, depending on their exposure to a CO_2_-rich atmosphere and the water-to-cement ratio. Std. Dvtn “means standard deviation” and Coef. Var. (%) “means coefficient of variation (%)”.

	Mg/Si			
	C-1-H	NC-1-H	C-1-L	NC-1-L
Mean	0.85	1.35	1.27	1.18
Std. Dvtn	0.12	0.16	0.17	0.13
Coef. Var.(%)	14.72	12.22	13.08	10.60

**Table 2:** Characteristics of the raw materials used for the cement composition. Oxide composition, bulk density, surface area, and particle size distribution were determined by X-ray fluorescence (XRF), helium pycnometry, the BET method, and laser diffraction, respectively. LOI stands for loss on ignition. “***” means that there is no presence of the indicated elements in the table.

	Composition (Weight %)								Bulk Density	Surface Area	Particle Size		
											D_10_	D_50_	D_90_
	MgO	SiO_2_	CaO	Fe_2_O_3_	Al_2_O_3_	Cl^−^	SO_3_^−^	LOI	(g/cm^3^)	(m^2^/g)	(µm)	(µm)	(µm)
MgO	97.5	0.22	0.6	0.08	0.1	0.34	0.04	1.1	0.35	21	1.69	6.25	15.7
SiO_2_	0.3	95.7	0.35	0.25	0.65	***	***	1.9	0.55	26	0.1	0.7	12.01

**Table 3:** Main features of each of the formulations used in the test pastes. “NC” denotes non-carbonated samples, while “C” denotes carbonated samples. The numbers “0”, “0.25”, and “1” represent the different SiO_2_/MgO ratios employed. “L” indicates lower water demand, while “H” indicates higher water demand. * CO_2_ refers to exposure to a saturated atmosphere of this gas for 72 h. For samples analyzed at 7 days, the exposure occurs at 4 days of age, while for those analyzed at 28 days, it occurs at 25 days of age.

Mix Denotation	SiO_2_/MgO (By Weight)	Water/solid (By Weight)	CO_2_ * Exposure
NC-0-L	0	0.42	No
C-0-L	0	0.42	Yes
NC-0-H	0	0.8	No
C-0-H	0	0.8	Yes
NC-0.25-L	0.25	0.42	No
C-0.25-L	0.25	0.42	Yes
NC-0.25-H	0.25	0.8	No
C-0.25-H	0.25	0.8	Yes
NC-1-L	1	0.42	No
C-1-L	1	0.42	Yes
NC-1-H	1	0.8	No
C-1-H	1	0.8	Yes

**Figure A1:**
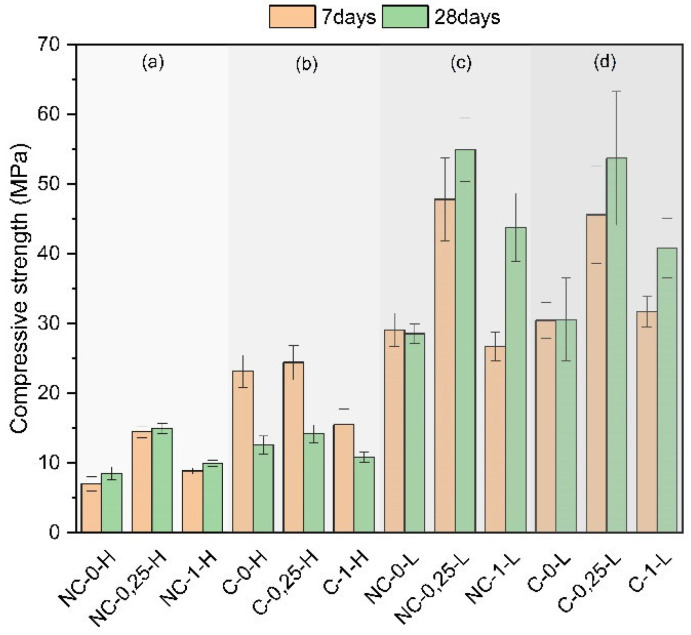
Bar chart displaying the compressive strength values (average and standard deviation) of the studied formulations based on the results shown in Table 1.

**Figure A2:**
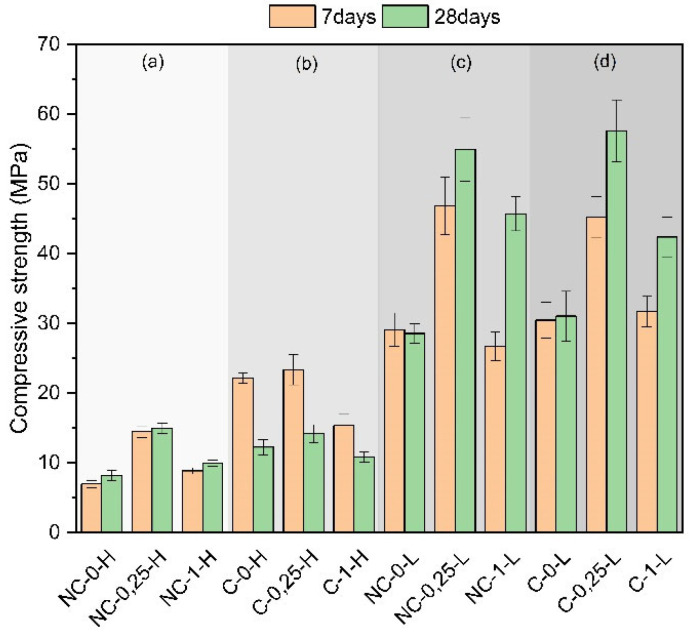
Bar chart displaying the compressive strength values (mean and standard deviation) for the various formulations studied, based on the results shown in Table A2, following the removal of all outliers according to the aforementioned criteria.

## Data Availability

Data is contained within the article (and Supplementary Materials).

## Supplementary Materials

The following supporting information can be downloaded at https://www.mdpi.com/article/10.3390/molecules30051072/s1, Figure S1: Complete diffractograms of the analyzed samples: (a) non-carbonated and (b) carbonated at 7 days of age.; Figure S2: Complete diffractograms of the analyzed samples: (a) non-carbonated and (b) carbonated at 28 days of age.

## Appendix A

Mechanical tests were conducted on prismatic samples measuring 1 × 1 × 6 cm^3^. Six samples were tested for each formulation. Average values ($\bar{x}$) and standard deviations (σ) were calculated for each group. To ensure the comparison of normally distributed populations and mitigate errors associated with the small sample size, the coefficient of variation CV(%) was determined according to equation (Equation (A1)).

(A1) $$CV\left(\%\right)=\frac{\bar{x}}{\sigma }\times 100\%$$

Outliers were progressively excluded when the CV(%) exceeded 10% to achieve populations with homogenous result dispersion. The exclusion criterion involved removing values that deviated the most from the mean, with a maximum of two exclusions to maintain a sufficient sample size.

Columns in black represent data meeting the criterion of a coefficient of variation below 10%, while those in red do not. Although two formulations did not meet this criterion even after removing up to two outliers, the coefficient of variation remained around 11%, which is statistically acceptable.

**Table A1:** Raw, unprocessed results for each formulation at 7 days (top of the table) and 28 days (bottom of the table).

	NC- 0- H	NC-0.25-H	NC- 1- H	C- 0- H	C-0.25-H	C- 1- H	NC- 0- L	NC-0.25- L	NC- 1- L	C- 0- L	C-0.25-L	C- 1- L
1	5.52	13.86	8.75	21.10	21.80	12.74	31.74	41.42	28.34	27.21	35.05	28.19
2	6.52	13.55	8.54	21.69	22.98	13.46	32.08	42.98	28.36	33.93	41.66	30.17
3	6.78	16.11	8.88	22.02	25.44	14.60	29.77	45.04	25.39	28.55	43.58	33.73
4	6.79	14.71	9.80	22.88	26.10	15.42	28.70	45.72	23.31	32.52	46.02	30.76
5	7.86	14.09	8.78	23.09	23.96	18.02	25.86	53.73	29.05	28.26	49.64	34.45
6	8.76	14.56	8.47	28.11	19.74	18.76	26.45	57.91	25.81	32.29	57.77	32.84
$\bar{x}$	7.04	14.48	8.87	23.15	23.34	15.50	29.10	47.80	26.71	30.46	45.62	31.69
σ	1.03	0.83	0.44	2.32	2.15	2.22	2.38	5.96	2.04	2.54	7.02	2.18
CV(%)	14.63	5.73	4.96	10.02	9.23	14.32	8.18	12.47	7.64	8.34	15.39	6.88
	NC- 0- H	NC-0.25-H	NC- 1- H	C- 0- H	C-0.25-H	C- 1- H	NC- 0- L	NC-0.25- L	NC- 1- L	C- 0- L	C-0.25-L	C- 1- L
1	7.06	16.32	10.14	11.07	13.53	9.88	28.07	62.58	34.03	20.80	34.36	33.21
2	7.87	14.31	10.37	11.15	16.19	12.17	28.96	56.17	42.81	27.89	49.46	38.71
3	8.32	15.31	9.24	12.02	13.35	10.14	26.93	51.34	43.97	28.05	56.44	40.35
4	8.64	14.20	9.58	13.18	12.59	10.89	26.78	54.34	44.75	31.56	59.55	42.04
5	9.19	14.95	10.41	13.81	15.71	11.03	30.22	57.26	47.74	36.65	60.37	43.97
6	9.98	14.48	10.20	14.37	13.64	10.81	30.28	48.07	49.38	38.58	62.09	46.82
$\bar{x}$	8.51	14.93	9.99	12.6	14.17	10.82	28.54	54.96	43.78	30.59	53.71	40.85
σ	0.93	0.73	0.43	1.27	1.31	0.73	1.41	4.58	4.90	5.93	9.56	4.28
CV(%)	10.93	4.89	4.30	10.08	9.24	6.75	4.94	8.33	11.19	19.39	17.80	10.48

**Table A2:** Results after the removal of the first outlier (if necessary) for each formulation at 7 days (top of the table) and 28 days (bottom of the table).

	NC- 0- H	NC-0.25-H	NC- 1- H	C- 0- H	C-0.25-H	C- 1- H	NC- 0- L	NC-0.25- L	NC- 1- L	C- 0- L	C-0.25- L	C- 1- L
1	5.52	13.86	8.75	21.10	21.80	13.46	31.74	42.98	28.34	27.21	41.66	28.19
2	6.52	13.55	8.54	21.69	22.98	14.60	32.08	45.04	28.36	33.93	43.58	30.17
3	6.78	16.11	8.88	22.02	25.44	15.42	29.77	45.72	25.39	28.55	46.02	33.73
4	6.79	14.71	9.80	22.88	26.10	18.02	28.70	53.73	23.31	32.52	49.64	30.76
5	7.86	14.09	8.78	23.09	23.96	18.76	25.86	57.91	29.05	28.26	57.77	34.45
6		14.56	8.47		19.74		26.45		25.81	32.29		32.84
$\bar{x}$	6.70	14.48	8.87	22.16	23.34	16.05	29.10	49.08	26.71	30.46	47.73	31.69
σ	0.75	0.83	0.44	0.74	2.15	1.74	2.38	5.73	2.04	2.54	5.68	2.18
CV(%)	11.17	5.73	4.96	3.35	9.23	10.81	8.18	11.68	7.64	8.34	11.91	6.88
	NC- 0- H	NC-0.25-H	NC- 1- H	C- 0- H	C-0.25-H	C- 1- H	NC- 0- L	NC-0.25- L	NC- 1- L	C- 0- L	C-0.25- L	C- 1- L
1	7.06	16.32	10.14	11.07	13.53	9.88	28.07	62.58	42.81	27.89	49.46	38.71
2	7.87	14.31	10.37	11.15	16.19	12.17	28.96	56.17	43.97	28.05	56.44	40.35
3	8.32	15.31	9.24	12.02	13.35	10.14	26.93	51.34	44.75	31.56	59.55	42.04
4	8.64	14.20	9.58	13.18	12.59	10.89	26.78	54.34	47.74	36.65	60.37	43.97
5	9.19	14.95	10.41	13.81	15.71	11.03	30.22	57.26	49.38	38.58	62.09	46.82
6		14.48	10.20		13.64	10.81	30.28	48.07				
$\bar{x}$	8.2158	14.93	9.99	12.25	14.17	10.82	28.54	54.96	45.73	32.55	57.58	42.38
σ	0.7202	0.73	0.43	1.089	1.31	0.73	1.41	4.58	2.45	4.38	4.46	2.83
CV(%)	8.77	4.89	4.30	8.89	9.24	6.75	4.94	8.33	5.35	13.47	7.74	6.67

**Table A3:** Results after the removal of the second outlier (if necessary) for each formulation at 7 days (top of the table) and 28 days (bottom of the table).

	NC- 0- H	NC-0.25-H	NC- 1- H	C- 0- H	C-0.25-H	C- 1- H	NC- 0- L	NC-0.25- L	NC- 1- L	C- 0- L	C-0.25- L	C- 1- L
1	6.52	13.86	8.75	21.10	21.80	13.46	31.74	42.98	28.34	27.21	41.66	28.19
2	6.78	13.55	8.54	21.69	22.98	14.60	32.08	45.04	28.36	33.93	43.58	30.17
3	6.79	16.11	8.88	22.02	25.44	15.42	29.77	45.72	25.39	28.55	46.02	33.73
4	7.86	14.71	9.80	22.88	26.10	18.02	28.70	53.73	23.31	32.52	49.64	30.76
5		14.09	8.78	23.09	23.96		25.86		29.05	28.26		34.45
6		14.56	8.47		19.74		26.45		25.81	32.29		32.84
$\bar{x}$	6.99	14.48	8.87	22.16	23.34	15.38	29.10	46.87	26.71	30.46	45.23	31.69
σ	0.52	0.83	0.44	0.74	2.15	1.68	2.38	4.09	2.04	2.54	2.98	2.18
CV(%)	7.39	5.73	4.96	3.35	9.23	10.92	8.18	8.72	7.64	8.34	6.60	6.88
	NC- 0- H	NC-0.25-H	NC- 1- H	C- 0- H	C-0.25-H	C- 1- H	NC- 0- L	NC-0.25- L	NC- 1- L	C- 0- L	C-0.25- L	C- 1- L
1	7.06	16.32	10.14	11.07	13.53	9.88	28.07	62.58	42.81	27.89	49.46	38.71
2	7.87	14.31	10.37	11.15	16.19	12.17	28.96	56.17	43.97	28.05	56.44	40.35
3	8.32	15.31	9.24	12.02	13.35	10.14	26.93	51.34	44.75	31.56	59.55	42.04
4	8.64	14.20	9.58	13.18	12.59	10.89	26.78	54.34	47.74	36.65	60.37	43.97
5	9.19	14.95	10.41	13.81	15.71	11.03	30.22	57.26	49.38		62.09	46.82
6		14.48	10.20		13.64	10.81	30.28	48.07				
$\bar{x}$	8.2158	14.93	9.99	12.25	14.17	10.82	28.54	54.96	45.73	31.04	57.58	42.38
σ	0.7202	0.73	0.43	1.089	1.31	0.73	1.41	4.58	2.45	3.56	4.46	2.83
CV(%)	8.77	4.89	4.30	8.89	9.24	6.75	4.94	8.33	5.35	11.46	7.74	6.67
