# Supplementary material for: Carbonation and Phase Evolution in MgO-SiO2 Cements: Impact on Strength
Source: Molecules. 2025 Feb 26;30(5):1072. doi: 10.3390/molecules30051072 (PMC11902274; doi:10.3390/molecules30051072)
Supplement: Supplementary file 1 [file molecules-30-01072-s001.zip › molecules-3462351-supplementary.pdf]

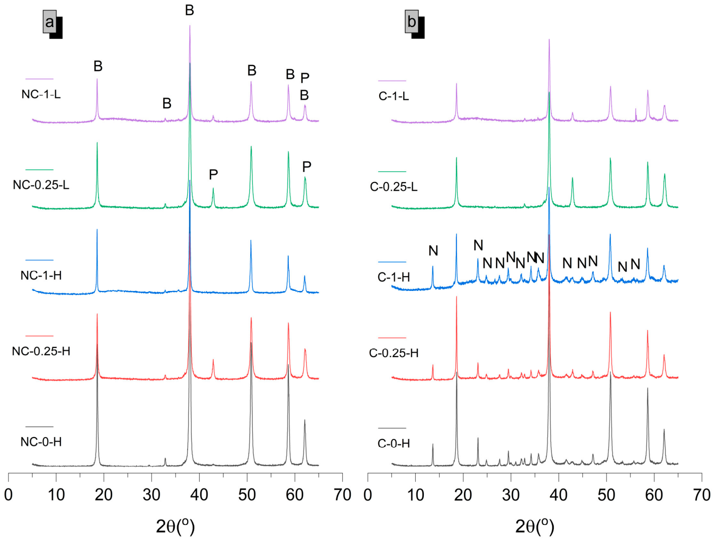

Figure S1-Complete diffractograms of the analyzed samples: (a) non-carbonated and (b) carbonated at 7 days of age.

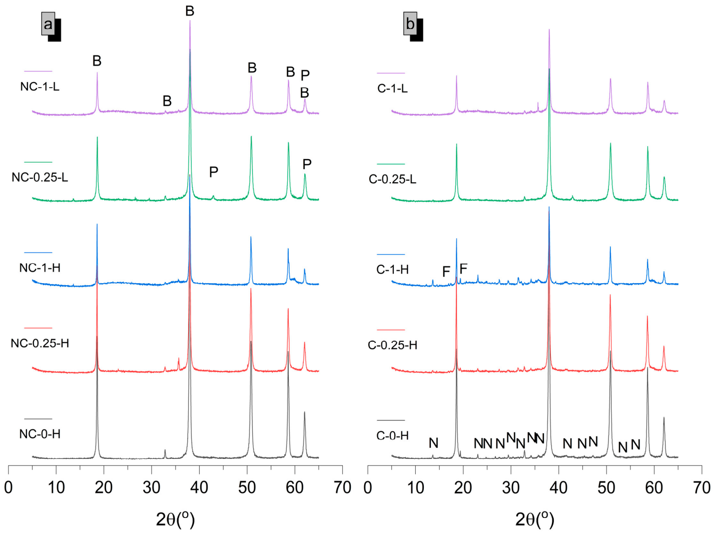

Figure S2- Complete diffractograms of the analyzed samples: (a) non-carbonated and (b) carbonated at 28 days of age.
